# Supplementary material for: Liver-Targeted Scutellarin Nanoemulsion Alleviates Fibrosis with Ancillary Modulation of the Gut–Liver Microbiota
Source: Int J Mol Sci. 2025 Oct 7;26(19):9746. doi: 10.3390/ijms26199746 (PMC12524853; doi:10.3390/ijms26199746)
Supplement: Supplementary file 1 [file ijms-26-09746-s001.zip › ijms-3830583-supplementary.pdf]

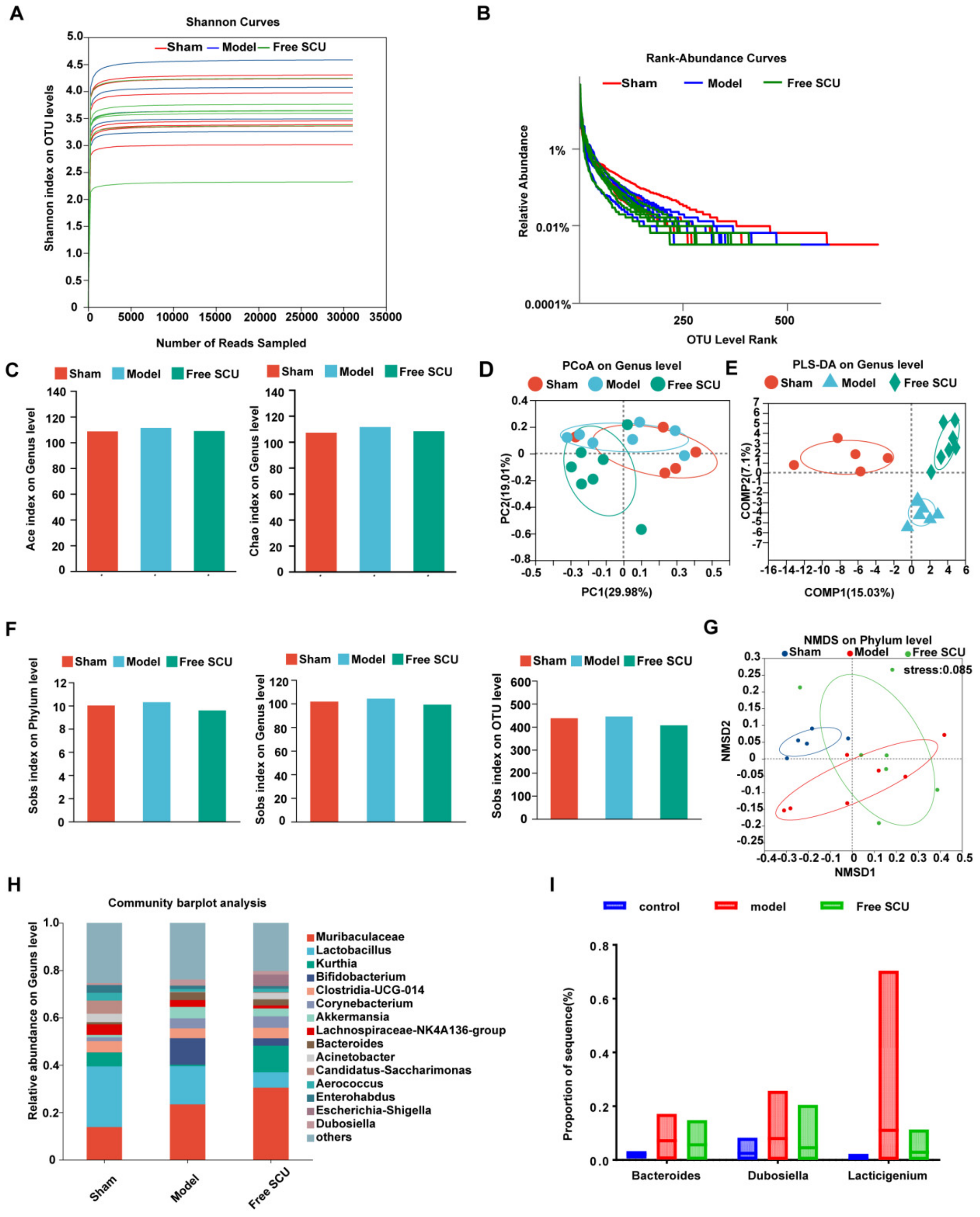

Figure S1: SCU improved the disturbance in the gut microbiota. (A) Shannon rarefaction curves of observed taxonomic units. (B) rank-abundance of observed taxonomic units. (C) Alpha diversity at Genus level quantified by Ace and Chao index. (D) Principal Coordinates Analysis at Genus level, (E) Partial Least Squares Discriminant Analysis at Genus level. (F) Alpha diversity at Phylum, Genus and

OTU level quantified by Sobs index. (G) Non-metric Multidimensional Scaling at Phylum level. (H) Relative Abundance on Genus level. (I) A genus of bacteria improved by SCU at the generic level.

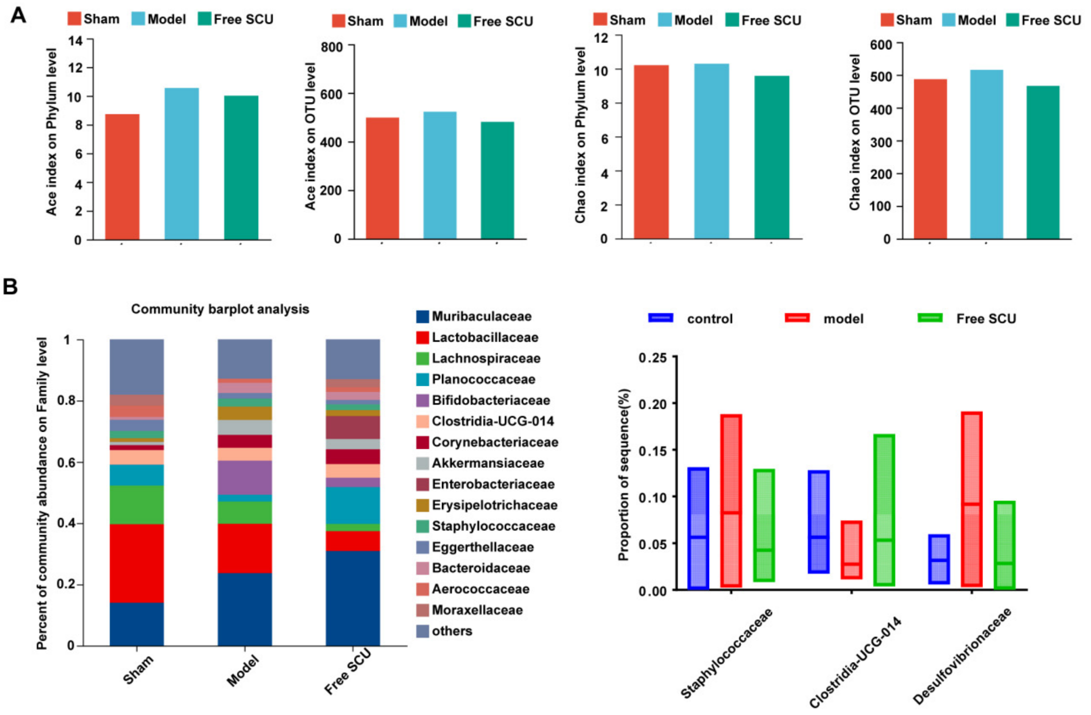

Figure S2: (A) Alpha diversity indices (ACE and Chao1) at the phylum and OTU levels for Sham, BDL, and SCU-treated groups. (B) Relative abundance of selected bacterial families in the gut.

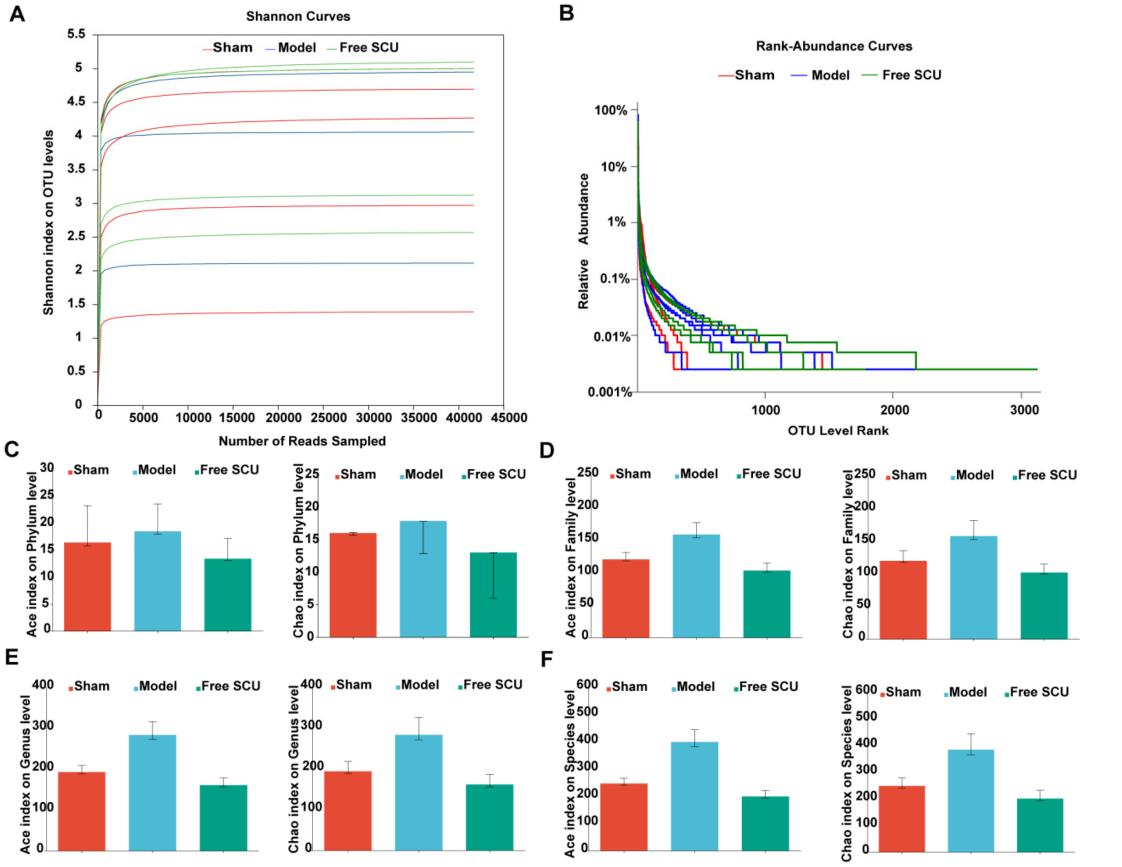

Figure S3: (A) Shannon rarefaction curves of observed taxonomic units and rank-abundance of observed taxonomic units. (C-F) Alpha diversity (ACE and Chao1 richness indices) at the phylum (C), family (D), genus (E), and species (F) levels for liver microbiota.
